# The AIDS pandemic in the 2020s: community responses bring female underserved populations into sharper focus

**DOI:** 10.1002/jia2.25745

**Published:** 2021-06-30

**Authors:** Lilian Mworeko, Ntando Yola, Daouda Diouf, Linda‐Gail Bekker

**Affiliations:** ^1^ International Community of Women living with HIV Eastern Africa (ICWEA) Wakiso District Uganda; ^2^ Advocacy for Prevention of HIV and AIDS Johannesberg South Africa; ^3^ The Desmond Tutu HIV Centre Faculty of Health Sciences University of Cape Town Cape Town South Africa; ^4^ Enda Santé Dakar Senegal

**Keywords:** HIV/AIDS, Community‐based programming, Gender and sexually diverse populations, HIV services / HIV service provision, sub‐Saharan Africa, Human rights approach

The UNAIDS targets seek to contain the HIV epidemic by 2030, yet globally the 2020 milestones were missed [1, 2]. While some regions performed well, boosted by strong multisectoral leadership, committed resources, community mobilization and approaches based on scientific evidence and human rights, others were off track even before the COVID‐19 pandemic struck in January 2020 [1, 2]. Unfortunately, the direct and indirect effects of the COVID‐19 pandemic are already showing further impact on HIV outcomes, with a predicted loss of gains made in the last two decades [2]. COVID‐19 further catapaulted into sharp focus the global health inequalities that continuously undermine progress in HIV and health responses generally [3]. Such inequalities manifest as poor access to information, inadequate care and treatment and absent screening and prevention services, especially for those that find mainstream services inaccessible [4].

Community programmes are vital in reaching marginalized populations yet funding is unstable and is threatened by emergent competing priorities, as evidenced in the last year. The total resources available for HIV programmes in low‐ and middle‐income countries waned in 2020, with financial resources from international for the HIV responses declining by nearly 10% since 2015, with domestic funding additionally starting to decline [1, 5]. Components of national HIV programmes that cater to key populations often depend heavily on external funding and so are at particular risk during times of constrained resources and funding transitions as has been seen recently in middle‐income countries [6, 7]. The turmoil associated with COVID‐19 presents all countries with difficult resource allocation decisions. Community‐based programmes have been and will continue to be negatively affected by such decision making [5]. This pattern of funding reallocation undermines more sustained strengthening of the health system and integrated, community care as a whole. In addition, it is likely that more resources rather than less will be needed to win back any progress in the HIV response lost in 2020 during the COVID‐19 pandemic.

Underserved and marginalized “key” populations at high risk of HIV infection, such as those who sell sex, use drugs or who belong to sexually and gender diverse groups, require particular focus as they face intersectional health and social challenges with continued stigma and discrimination, and poor access to health services. Key populations are predicted to contribute more substantially to HIV incidence and morbidity in sub‐Saharan Africa in the coming years. Female members of key populations face additional barriers and unique risk factors [4]. The HIV response showed us that integrated services that use community‐led and rights‐based peer‐led approaches, can effectively improve service coverage, health outcomes and reduce health inequalities [8]. Recent community responses in the African region to HIV alone and alongside COVID‐19 illustrate that the unique needs of key populations can successfully be matched at the community level. If such responses are adequately supported and funded, community‐based initiatives may enhance engagement in care among diverse populations.

Female sex workers (FSW) require a rights‐based approach that understands the size, characteristics and needs of this population locally. In 2009 in Zimbabwe, Sisters with a Voice was established as a comprehensive, evidence‐driven programme with the aim to foster an empowered and resilient FSW community fully engaged in HIV prevention and care cascades. The programme provided ten static and 26 outreach sites tailored to FSW needs, nested within public sector facilities. Full‐ or part‐time sex workers throughout Zimbabwe, with an emphasis on hotspots at border posts and major transport routes, were included in the programme. Through intensification and scale‐up, thousands of formal and informal FSW were reached, improving linkage and retention in care [9]. The programme was found to be highly acceptable to FSW and providers, attracting higher numbers of new FSW, performing more than double the number of HIV tests, as well as double the number of diagnoses. Antiretroviral uptake improved, whereas reports of HIV‐related stigma declined. Despite this success, overall viral load suppression rates among FSW did not improve compared to FSW attending standard of care facilities, and it is expected that more sustained efforts are required to achieve this desirable outcome [9].

Women who inject and use drugs face an intersectional burden of hardship, including wholescale criminalization of drug use with the added stigmatization imposed on women who use drugs, the frequent loss of custody of children that results, and the potential for increased exposure to gender‐based violence [4]. A recent report by the International AIDS Society highlighted a number of community‐based organizations engaged in bringing tailored services and much needed care to these populations. One example is the Harm Reduction Community Container project, which works to identify, diagnose and link women who inject drugs in Mauritius to care. Developed by Collectif Urgence Toxida, it blends community‐based healthcare services (including HIV and hepatitis care and counselling, safe injecting practices and clean apparatus and condoms) using mobile‐equipped container units for people who are reluctant to attend traditional facilities [10]. Such integrated healthcare services for key populations can ensure access to holistic care, as opposed to the norm where healthcare and harm reduction programmes remain siloed if they even do exist.

Populations that are vulnerable to HIV have been shown to be similarly vulnerable to COVID‐19. COVID‐19‐associated lockdowns negatively affected key populations with limitations in treatment access, prevention, sexual and reproductive health services (SRHS), as well as the increased potential for forced HIV status disclosure and loss of income. Such populations have been shown to struggle against the triple impact of HIV, COVID‐19 and poverty. In West Africa, Enda Santé, a Senegalese nongovernmental organization that provides HIV care across diverse populations negotiated a re‐allocation of funds to support COVID‐19‐related activities in the most‐affected communities, including those with high poverty levels, severe overcrowding and inadequate healthcare provision. They mobilized and trained young and female community leaders to go door to door and deliver health education, diagnosis and linkage to care [11]. In Uganda, several community‐based organizations serving people living with HIV and disabilities collectively secured resources, food and other items to support over 5,000 people. In another example, the International Community of Women living with HIV Eastern Africa (ICWEA) distributed 55 bicycles to facilitate peers with treatment and prevention drug refills, SRHS, as well as psychosocial support through home visits and toll‐free helplines for counselling and information. The efforts to maintain HIV and other health services during COVID‐19 lockdowns underscore the value of differentiated services, including community and peer‐led services that are grounded in lived realities and responsive to the needs, priorities and rights of populations most burdened by HIV but which are without mainstream services. As new pandemics are expected to emerge and present challenges to public health, effective means of integrating HIV responses within broader responses will benefit current as well as future responses [12].

Community and peer‐based approaches are necessary if key populations are to be effectively reached, which presents a growing concern. The HIV epidemic has taught us and the COVID‐19 pandemic has underlined that in order to reach these populations we must continuously and sustainably invest in grassroots, peer‐ and community‐led organizations. If we do not, priority setting will be misplaced and mismatched to actual need, with the risk of wasting the limited resources available.

## Competing interests

The authors have declared no conflict of interest.

## Authors’ contributions

LBG drafted the initial manuscript. LM, NY, DD reviewed, provided input to and approved the final manuscript.
